# Tc-99m TRODAT-1 SPECT Is a Potential Biomarker for Restless Leg Syndrome in Patients with End-Stage Renal Disease

**DOI:** 10.3390/jcm9030889

**Published:** 2020-03-24

**Authors:** Yi-Chou Hou, Yu-Ming Fan, Ya-Ching Huang, Ruei-Ming Chen, Cheng-Hsu Wang, Yi-Te Lin, Tzung-Hai Yen, Kuo-Cheng Lu, Yuh-Feng Lin

**Affiliations:** 1 Graduate Institute of Clinical Medicine, College of Medicine, Taipei Medical University, Taipei 110, Taiwan; athletics910@gmail.com; 2 Department of Internal Medicine, Cardinal Tien Hospital, New Taipei City 231,Taiwan; lizarddad@gmail.com; 3 School of Medicine, Fu Jen Catholic University, New Taipei City 242, Taiwan; ymfan88@gmail.com; 4 Department of Nuclear Medicine, Cardinal Tien Hospital, New Taipei City 231,Taiwan; ck.dan120@gmail.com; 5 Department of Laboratory Medicine, Chang Gung Memorial Hospital, Linkou, Taoyuan 333, Taiwan; hycymm@cgmh.org.tw; 6 Department of Medical Biotechnology and Laboratory Science, College of Medicine, Chang Gung University, Taoyuan 333, Taiwan; 7 Graduate Institute of Medical Sciences, College of Medicine, Taipei Medical University, Taipei 110, Taiwan; rmchen@tmu.edu.tw; 8 School of Biomedical Engineering and Environmental Science, National Tsing Hua University, Hsinchu City 300, Taiwan; 9 Departments of Hyperbaric Medicine and Neurology, Cardinal Tien Hospital, New Taipei City 231, Taiwan; 10 Division of Nephrology, Chang Gung Memorial Hospital, Taipei 105, Taiwan; m19570@adm.cgmh.org.tw; 11 College of Medicine, Chang Gung University, Taoyuan 333, Taiwan; 12 Department of Nephrology, Fu Jen Catholic University Hospital, New Taipei City 243, Taiwan; 13 Department of Nephrology, Taipei Tzu Chi Hospital, Buddhist Tzu Chi Medical Foundation, New Taipei City 231, Taiwan; 14 Division of Nephrology, Department of Internal Medicine, Shuang Ho Hospital, Taipei Medical University, New Taipei City 235, Taiwan; 15 Graduate Institute of Medical Sciences, National Defense Medical Center, Taipei 114, Taiwan

**Keywords:** restless leg syndrome, ESRD, TRODAT-1

## Abstract

Rationales: Restless leg syndrome (RLS) is a common complication in patients with end-stage renal disease (ESRD). However, there is a lack of biomarkers linking uremic RLS to dopaminergic neurons. Previous studies demonstrated that Tc-99m TRODAT-1 SPECT was a biomarker for RLS but the correlation between the physiologic parameter was lacking. Methods: Overall, 32 patients were enrolled in the study and divided into the following 3 groups: (1) control (*n* = 13), (2) ESRD without RLS (*n* = 8) and (3) ESRD with RLS (*n* = 11). All patients had a clinical diagnosis of RLS and received Tc-99m TRODAT-1 SPECT. A subgroup analysis was performed to compare differences between the control and ESRD with RLS groups. Tc-99m TRODAT-1 SPECT was performed and activities in the striatum and occipital areas were measured using manually delineated regions of interest (ROIs) by an experienced nuclear medicine radiologist who was blinded to clinical data. Results: The total ratio of Tc-99m TRODAT SPECT was lower in the ESRD with RLS group (*p* = 0.046). The uptake ratio of TRODAT negatively correlated with serum parathyroid hormone (*r* = −0.577, *p* = 0.015) and ferritin (*r* = −0.464, *p* = 0.039) concentrations. However, the uptake positively correlated with the hemoglobin concentration (*r* = 0.531, *p* = 0.011). The sensitivity and specificity of the total TRODAT ratio for predicting RLS in the overall population were 95.0% and 67.7%, respectively, at a cutoff value of 0.980 (area under the curve of receiver operating characteristic curve was 0.767, *p* = 0.024). Conclusion: In patients with ESRD and RLS, Tc-99m TRODAT might be a potential biomarker. Dysregulated hemoglobin, serum parathyroid hormone and serum ferritin concentrations might influence the uptake of the TRODAT ratio.

## 1. Introduction

Chronic kidney disease (CKD), defined as chronic renal function impairment because of the loss of glomerular filtration for more than 3 months, is a global health issue owing to its multiple complications and comorbidities [1]. With the progression of glomerular filtration loss, the dysregulated fluid balance system, hormonal activation and specific uremic toxin retention induce systemic diseases, such as cardiovascular disease, renal osteodystrophy, dysregulated immunity and neurologic disorders [2]. Uremia-related encephalopathy can be categorized according to disease chronicity. In acute uremic encephalopathy, the acute retention of urea nitrogen and the imbalance of serum sodium and glucose concentrations alter consciousness and CKD severity and acute uremic encephalopathy may be corrected through renal replacement therapy. However, chronic neurologic complications, such as peripheral neuropathy, cognitive dysfunction or memory deficit, Parkinsonism and restless leg syndrome (RLS), are common in patients with CKD. Neurologic complications in patients with CKD are associated with limited activities of daily life and depressive mood, which are linked to devastating comorbidities, such as infection or increased mortality [3]. Therefore, it is imperative to recognize risk factors and biomarkers for these neurologic complications in patients with CKD.

RLS is a sensorimotor disorder that occurs during inactivity or at rest and worsens in the evening and night. The key features of RLS are sensory symptoms, namely restlessness (urge to move) and unpleasant sensations (paresthesia and pain) and motor symptoms (periodic limb movements and other motor manifestations). Because RLS aggravates during rest or sleep, it affects the quality of sleep, thereby leading to multiple comorbidities [4]. Primary or idiopathic RLS is highly associated with familial and genetic risk factors and secondary RLS is associated with iron deficiency anemia, multiple sclerosis, Parkinson’s disease and hypertension [5]. Notably, individuals with end-stage renal disease (ESRD) or chronic renal insufficiency are susceptible to secondary RLS. The prevalence of RLS was approximately 10–15% in the general population, whereas the incidence of RLS was even higher in patients undergoing hemodialysis (6–60%) [6]. Several risk factors are linked to uremic RLS, including anemia, hyperphosphatemia, secondary hyperparathyroidism, hypertension and diabetes mellitus. In ESRD patient with RLS, the period of dependence for dialysis was longer and RLS could induce sleep fragmentation and sleep deprivation, which is linked to cardiovascular disease at the same time [7]. Notably, aberrant metabolism of iron in the brain and dysregulation of central dopaminergic neurotransmission are typically observed in patients with uremic RLS [8]. Furthermore, the declining glomerular filtration rate dysregulates the homeostasis of elementary heavy metals. Consequently, the burden of heavy metals, such as copper and nickel, is increased in the body of patients with CKD or ESRD. Previous studies have revealed that copper can directly affect the dopaminergic neuron function in patients without renal function impairment. In addition, in patients with CKD, dysregulated heavy metal metabolism is related to high cardiovascular comorbidities. However, there is ambiguity regarding the correlation between dysregulated heavy metals and neurologic disorders in patients with CKD.

To study the functional imaging of the dopaminergic system in RLS patients, radioactive isotopes can be applied to directly evaluate receptors in dopaminergic neurons or regional cerebral blood flow distribution [9]. Radioligands focusing on the dopamine transporter (DAT) have been used in neuroimaging for brain disorders associated with dopaminergic degeneration [10]. Several studies had applied presynaptic dopamine tracer to diagnose idiopathic RLS, such as N-((E)-3-iodopropen-2-yl)-2 beta-carbomethoxy-3 beta-(4-chlorophenyl) tropane ([123I]IPT—single photon emission computed tomography (SPECT) or 18Fluoro-L-Dopa (F-DOPA) Positron Emission Tomography (PET) but the result for diagnosing RLS was controversial [11]. A recent study suggested that RLS is associated with decreased DAT binding potential. Notably, technetium-99 m-[2-[[2-[[[3-(4-chlorophenyl)-8-methyl-8-azabicyclo(3,2,1)oct-2-yl]methyl] (2-mercaptoethyl)amino]ethyl]amino]ethanethiolato(3-)-N2,N2ʹ,S2,S2ʹ]oxo-[1R-(exo-exo)] (Tc-99m TRODAT) is a radiolabeled DAT tracer that selectively binds to DAT and has an excellent brain uptake after injection [12,13]. DAT imaging using Tc-99m TRODAT-1 could elucidate the reduction of dopaminergic agent uptake in several neurological diseases. Moreover, in our previous study, Tc-99m TRODAT served as a biomarker in patients with RLS [14]. Tc-99m TRODAT is considered as a cost-effective neuroimaging agent without pharmacological side effects. Nonetheless, because RLS is associated with dysregulation of the dopaminergic system, the application of TRODAT-1 in patients with uremia and RLS is still elusive, in addition to the lack of correlation of TRODAT-1 with other hematologic or biochemical parameters.

Current clinical evidence indicates that TRODAT-1 might reflect the dysfunction of the dopaminergic system in RLS and patients with uremia are susceptible to RLS. Therefore, this study elucidated the role of Tc-99m TRODAT-1 in patients with CKD and RLS as well as analyzed possible contributing biochemical and hematologic factors.

## 2. Materials and Methods

### 2.1. Ethics

This study was performed at a regional hospital in New Taipei City, Taiwan. All subjects gave their written informed consent for inclusion before they participated in the study. The study was conducted in accordance with the Declaration of Helsinki. The Ethics Committee on Human Studies at Cardinal Tien Hospital approved the study protocol (CTH-106-3-5-053). The study period was from 1 June 2018, to 20 June 2019. We obtained written informed consent from participants. Subsequently, patients were divided into 3 groups based on their characteristics. Patients’ demographic data were obtained from medical records in the hospital. Blood and urine sampling was performed. Tc-99m TRODAT-1 SPECT was arranged.

### 2.2. Study Design

After obtaining written informed consent, patients were divided into the following 3 groups—control, ESRD without RLS and ESRD with RLS. In the control group, patients who had an estimated glomerular filtration rate of >15 mL/min and did not have RLS were included. In the ESRD group, patients who had been receiving maintenance hemodialysis or peritoneal dialysis continuously for more than 3 months were included. In the ESRD with RLS group, patients who had been receiving maintenance hemodialysis or peritoneal dialysis continuously for more than 3 months with a 3-month diagnosis of RLS were included. The diagnosis of RLS was based on the following criteria reported in an international RLS study conducted in 2002 [15]: (1) an urge to move the limbs that is typically associated with paresthesia or dysesthesia, (2) symptoms that start or become worse with rest, (3) at least partial relief of symptoms with physical activity and (4) worsening of symptoms in the evening or at night (4). All patients had no previous diagnosis of cerebrovascular attack or Parkinsonism according to the medical record. Negative findings for bradykinesia, dyskinesia or other neurologic signs were confirmed by 2 trained physicians of the internal medicine department.

### 2.3. Demographic Data, Biochemical and Hematologic Results

Demographic data were obtained from medical records in Cardinal Tien Hospital. Hematologic and biochemical parameters were obtained after informed consent was obtained from patients. In patients with ESRD receiving HD, these parameters were obtained during the midday of the hemodialysis session before the hemodialysis treatment. Tc-99m TRODAT-1 SPECT protocolTc-99m TRODAT-1 SPECT was performed according to the protocol reported by Huang et al. [16]. Briefly, patients were asked to avoid high protein diet or medications influencing dopaminergic neurons, such as levodopa, 24 h before the TRODAT-1 analysis. The fan-beam collimator in Discovery NM 630 (GE Healthcare Inc.) was used for imaging. Each patient was administered 925 MBq (25 mCi) of Tc-99m TRODAT-1 through intravenous injection and imaging was performed approximately 3.5 h after injection. Patients were placed in the supine position and fixed with a head holder. Symmetrical windows with Tc-99m energy peaks of 140 keV ± 10% were used in a 360° circular orbit with rotation at 3° intervals for 30 s per angle step. Each projection data were collected for 20 s, for a total of 120 projections. The image reconstruction matrix size was 128 × 128 pixels, with a slice thickness of 1.99 mm. The Metz filter was used for filtered backprojection reconstruction; the cutoff frequency used for the filter with point spread was 9 and the power order was 5. Attenuation was corrected using the Chang method (attenuation coefficient μ = 0.12/cm). Activities in the striatum and occipital areas were measured using manually delineated regions of interest (ROIs) by an experienced nuclear medicine radiologist who was blinded to clinical data. ROIs were drawn over the whole striatum on composite images of 10 slices with highest basal ganglia activity. The size and shape of striatum ROI were used as a template in the occiput and the average values of pixels in individual ROI were calculated. The specific uptake ratio of the target area was determined using the following formula (1):

Ratio = (target minus reference) divided by reference(1)

The total uptake ratio of each participant was determined using the mean ratio from the bilateral caudate, putamen and basal ganglion.

### 2.4. Urinary Heavy Metal Analysis

The analysis of urinary heavy metals, including arsenic, cadmium, copper, lead, manganese, mercury, nickel and zinc, was performed as per the protocol reported by Huang et al. [17]. Briefly, 10 mL of the urine sample was obtained from patients. All patients were asked to avoid seafood 7 days before urine collection to avoid the influence of organic arsenic intake. The collected urine samples were stored in 10-mL decontaminated plastic collection tubes without metals. Eight heavy metals were quantified using inductively coupled plasma mass spectrometry (ICP-MS) on a PerkinElmer NexION 350Xinstrument (Waltham, MA, USA). Cadmium, copper and lead were analyzed using the no-gas mode. Dynamic reaction cell with methane was used to eliminate polyatomic interferences and quantify arsenic, manganese, nickel and zinc. Urine specimens (500 μL) were diluted (1:9) with a 1.5% nitric acid solution (JT Baker, Phillipsburg, NJ, USA) containing yttrium as the internal standard. Mercury was analyzed independently using the no-gas mode. Urine specimens (500 μL) were diluted (1:9) using ethylenediaminetetraacetic acid (0.04% *w*/*v*) (Wako, Richmond, VA, USA); ammonia solution (3.2% *v*/*v*) (*Shimakyu’s Pure* *Chemicals*, Osaka, Japan); 1-butanol (1.6% *v*/*v*) (Sigma, St. Louis, MO, USA); and triton X-100 (0.04% *v*/*v*) (Sigma, St. Louis, MO, USA) solutions with yttrium as the internal standard. All standards were purchased from High-Purity Standards (South Carolina, CA, USA). Calibration was performed after reagent blank and 6 calibration standards in the internal standard diluent solution described earlier. Calibration curves for all elements had an R ≥ 0.995.

### 2.5. Statistics

Continuous variables are expressed as the mean ± standard deviation and categorical values are expressed as the percentage. One-way ANOVA was applied to compare variables among the 3 groups. In the secondary analysis of the control and ESRD with RLS groups, student’s *t*-test was performed to compare differences in variables. Pearson’s correlation coefficient test was applied to compare the ratios of the Tc-99m TRODAT-1 and biochemical and hematological results, as well as the urinary heavy metal concentration. Receiver operating characteristic (ROC) curves were plotted and the area under the curve (AUC) was estimated to assess the predictive performance of the total TRODAT ratio. All statistical analyses were performed using the statistical package SPSS for Windows (Version XVII; SPSS, Inc., Chicago, IL, USA). A *p* value of less than 0.05 was considered significant.

## 3. Results

Table 1 demonstrates the demographic data of the 3 groups. The age of the control group was lower than that of the other 2 groups (*p* = 0.001). The percentage of diabetes mellitus was lower in the control group (*p* = 0.001). The percentage of medication use was similar among the 3 groups.

Table 2 presents the biochemical and hematologic results of the 3 groups. The serum blood urea nitrogen (BUN) and creatinine concentration were lower in the control group, which was compatible with the grouping design of the study. Regarding hematologic results, the serum hemoglobin concentration was higher in the control group than in the other 2 groups. The serum phosphorus concentration was lower in the control group.

**Figure 1:**
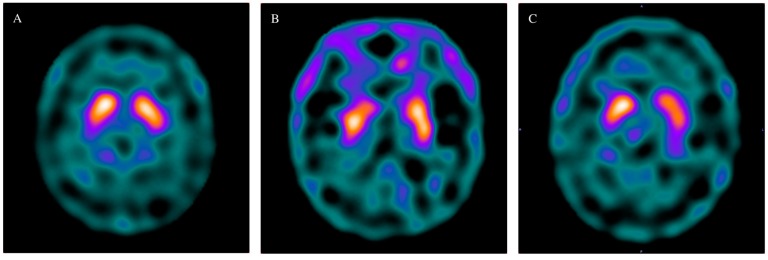
TRODAT ratio of the control (**A**), end stage renal disease (ESRD) without restless leg syndrome (RLS) (**B**) and ESRD with RLS (**C**) groups.

Figure 1 demonstrates the illustrations of patients in the control group (A), ESRD without RLS group (B) and ESRD with RLS group (C).

Table 3. demonstrates the TRODAT ratio of the 3 groups. Statistical differences were observed among the 3 groups in the total ratio (*p* = 0.046).

**Figure 2:**
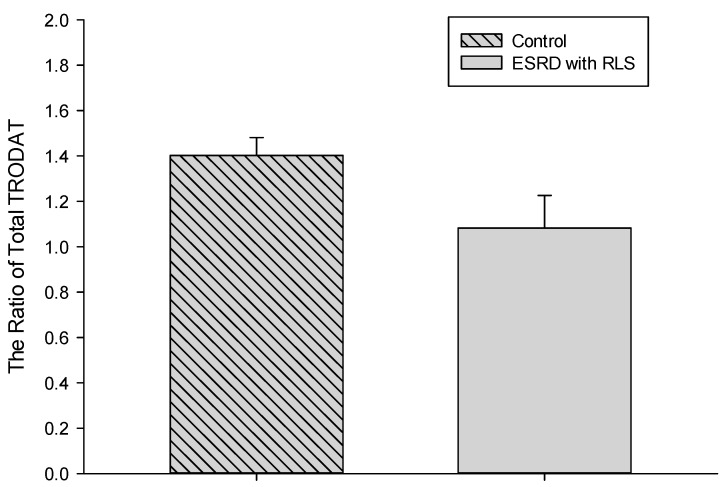
Comparative TRODAT ratio between the control and ESRD with RLS groups.

Figure 2 demonstrates the total ratio between the control and ESRD with RLS groups, with the ratio being lower in the ESRD with RLS group (1.06 ± 0.39 vs. 1.40 ± 0.28 in the control group, *p* = 0.025). The total ratio between ESRD with RLS and ESRD without RLS was similar (*p* = 0.235).

**Figure 3:**
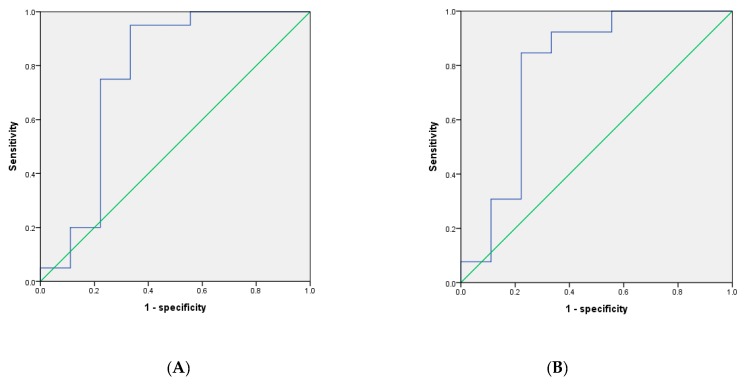
Receiver operating characteristic (ROC) curve for total TRODAT ratio as a predictor of RLS in the entire study population (**A**) and in the population including the control and ESRD with RLS groups (**B**).

The uptakes of specific regions were also determined. The uptakes in the right caudate (1.08 ± 0.36 vs. 1.47 ± 0.30 in the control group, *p* = 0.022) and right basal ganglion (1.04 ± 0.36 vs. 1.40 ± 0.27 in the control group, *p* = 0.011) were lower in the ESRD with RLS group than in the control group. Figure 3 demonstrates the diagnostic values of the total TRODAT ratio for the total study population and the population of the control and ESRD with RLS groups. The sensitivity and specificity of the total TRODAT ratio for predicting RLS in the overall population were 95.0% and 67.7%, respectively, at a cutoff value of 0.980 (AUC was 0.767, *p* = 0.024). By contrast, the sensitivity and specificity of the total TRODAT ratio for predicting RLS in the population of control and ESRD with RLS groups were 92.3% and 67.7%, respectively, at a cutoff value of 1.01 (AUC was 0.786, *p* = 0.025).

**Figure 4:**
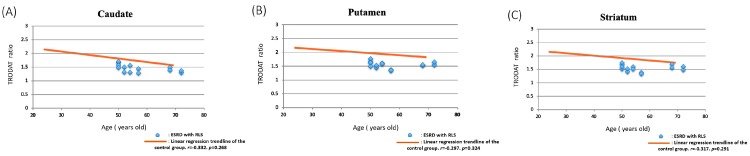
The linear regression of the caudate nucleus (**A**), putamen (**B**) and striatum (**C**) of the control group and the distribution of the ESRD with RLS group according to the age and the TRODAT ratio.

The linear regression between the age and the TRODAT ratio in caudate lobe, putamen and striatum of the control group and the distribution of ESRD with RLS patients were showed in Figure 4. The correlation between the age and the TRODAT ratio of the control group was −0.322 in the caudate nucleus (panel A, *p* = 0.288), −0.297 in the putamen (panel B, *p* = 0.324) and −0.317 in the striatum (panel C, *p* = 0.291). The distribution of TRODAT ratio in ESRD with RLS group were below the linear trendline of the control group at caudate nucleus, putamen and striatum.

Based on the results of one-way ANOVA and the ROC curve, we analyzed the correlation between the total TRODAT ratio and hematologic and biochemical parameters. Based on differences between the total TRODAT value between the control and ESRD with RLS groups, we compared the urinary concentration of heavy metals (Table 4). The urinary copper was higher in the RLS group (4.41 ± 1.92 μg/dL vs. 1.67 ± 1.02 μg/dL in the control group, *p* = 0.001). The urinary zinc concentration was lower in the RLS group (16.45 ± 21.92 μg/dL vs. 45.53 ± 29.92 μg/dL in the control group, *p* = 0.001).

Pearson’s correlation was performed to link the TRODAT ratio and hematologic and biochemical markers (Table 5). The correlation between TRODAT and age was 0.262 (not listed). The TRODAT ratio positively correlated with the serum hemoglobin concentration (*r* = 0.531, *p* = 0.011) and estimated glomerular filtration (*r* = 0.525, *p* = 0.007). However, the TRODAT ratio negatively correlated with the serum intact parathyroid hormone (*r* = −0.577, *p* = 0.015) and ferritin (*r* = −0.464, *p* = 0.039). Differences in other biochemical or hematologic parameters were not statistically significant.

## 4. Discussion

In our study, we enrolled 32 participants and they were divided into 3 groups, namely the control, ESRD without RLS and ESRD with RLS groups. We observed that the total TRODAT ratio was lower in the ESRD with RLS group than in the ESRD without RLS and control groups. Upon comparison of the ESRD with RLS and control groups, it was observed that the lower TRODAT ratio correlated with lower serum hemoglobin, higher serum BUN, higher intact parathyroid hormone, higher ferritin and higher urine nickel concentrations.

RLS is a disorder related to dopaminergic dysregulation and the treatment of RLS includes dopaminergic agents. By detecting blood flow or neuronal glucose uptake through PET or SPECT, previous studies have demonstrated decreased dopaminergic function in patients with ESRD who have psychiatric or neurologic disorders. The blood flow-based radioisotope detection by using Tc-99m ethyl cysteinate dimer (ECD) injection has demonstrated that dopaminergic uptake was disrupted in patients with ESRD who have depression [18]. However, no studies have detected presynaptic or postsynaptic dopaminergic receptors and our study is the first to demonstrate dysregulated dopaminergic function based on presynaptic neuronal uptake by using Tc-99m TRODAT-1. Our study results showed that the caudate and basal ganglion had statistically significant lower DAT density. Compared with other studies, the caudate lobe was observed to be a vulnerable area in patients with RLS upon performing functional studies [19,20]. Epidemiologic reports have revealed the association between CKD and dopaminergic disorders and CKD itself might worsen comorbidities, such as daytime sleepiness in Parkinson’s disease [21,22]. In our study, anemia negatively correlated with the DAT density. Epidemiologic studies have indicated that anemia is a potential risk factor for Parkinsonism or Parkinson’s disease [23,24]. Notably, iron deficiency is the most contributing factor owing to its dual effects—on the erythropoiesis and dopamine synthesis within the neurons [25]. In patients with ESRD, anemia is a common clinical problem because of the decreased production of erythropoietin and multiple comorbidities inducing the decrease in red blood cell production, such as iron deficiency or systemic inflammation [26]. In patients with ESRD, hypoxia-inducible factor (HIF) upregulation is contributed by anemia and HIF upregulation disturbs the dopamine release from dopaminergic cells [27]. Our study is the first to report the association between anemia and the function of the dopaminergic system through functional imaging. Compared with previous studies, our study observed that the plasma ferritin concentration inversely correlated with the TRODAT ratio. A possible explanation for this finding could be that the uptake of iron in the RBC of bone marrow decreased functionally because of inflammation or the effect of hepcidin [28,29]. Functional imaging studies have been performed in patients with CKD by using agents, such as ECD. Notably, ECD revealed that dysregulated blood flow was common in patients with CKD and such a result might reflect arterial stiffness that disrupts the energy demand in the brain [30]. Uremia and hyperphosphatemia are contributors to arterial stiffness in CKD [31] and the correlation of Tc-99m TRODAT-1 with the transcranial doppler scan might be a potential study goal to link the dysregulation of the dopaminergic system in CKD. Based on these findings, therapeutic strategies can focus on the correction of anemia aggressively in patients with uremic RLS and serial Tc-99m TRODAT-1 during anemia correction might be used to evaluate if RLS subsides.

In addition to anemia, the uptake of dopaminergic neurons negatively correlated with the serum parathyroid hormone in our study. Secondary hyperparathyroidism is common in patients with ESRD. The main contributing factor for secondary hyperparathyroidism is the “trade-off” theory of the decreased renal excretion of phosphate. A decrease in the glomerular filtration rate results in decreased renal excretion of phosphate, thereby activating phosphaturic hormone excretion, such as fibroblast growth factor 23 or parathyroid hormone, such that there is an increase in the reabsorption of phosphate through the sodium-phosphate cotransporter in remnant nephrons [32]. The parathyroid hormone reduces RBC formation by inhibiting their maturation in the bone marrow [33]. The abating effect on RBC might be a possible mechanism of hyperparathyroidism in RLS. Nevertheless, it is still ambiguous whether parathyroid hormone activates or inhibits dopaminergic neurons because most studies in the literature were case series [34]. However, several in vitro studies have demonstrated that the parathyroid hormone inhibits the activity of dopaminergic neurons in a dose-dependent manner [35]. Based on the previous literature, parathyroid hormone might influence the function of dopaminergic neurons directly or indirectly through the action of hemoglobin in patients with ESRD and such influence might contribute to RLS in these patients.

We observed that the urinary copper concentration and urinary copper-to-zinc ratio were higher in the ESRD with RLS group than in the control group. Dysregulated metabolism of trace elements is common in patients with ESRD [36]. In patients with CKD, the elimination of ingested elementary heavy metals would be less owing to impaired glomerular filtration and the dialysate might move or retain trace elements within the body [37]. In our study, the urinary copper concentration was higher in patients with RLS than in the control group, which is concordant with the findings of the systemic review conducted by Tonelli et al. [36]. The effect of copper toxicity on the neurologic system can be understood based on Wilson’s disease. Notably, excessive copper deposition within neurons disturbed cortical function and exacerbated dementia and excessive cerebral copper dysregulated dopaminergic neurons by influencing the MAT metabolism [38]. As per our results, the sensitivity and specificity of the total TRODAT ratio for predicting RLS in the control and ESRD with RLS groups were 100.0% and 91.8%, respectively, at a cutoff value of 2.50 (AUC was 0.955, *p* = 0.021, not demonstrated). However, no correlation was observed between the TRODAT ratio and the urinary copper concentration (*r* = −0.361, *p* = 0.141). One possible mechanism would be that the excessive copper concentration in the body is a contributor to anemia. Our results revealed that the urinary copper concentration negatively correlated with the hemoglobin concentration (*r* = −0.819, *p* = 0.001, not demonstrated). By contrast, the higher copper burden and higher copper to zinc ratio might be a biomarker for systemic inflammation [39], which worsens the severity of anemia. Although iron deficiency was proposed as a hypothesis of RLS, our data demonstrated that the serum ferritin concentration negatively correlated with the DAT density and the higher serum ferritin concentration might reflect the proinflammatory status [40]. The effect of copper and the regulation of the dopaminergic system might act through inflammatory activation and anemia. To decrease the copper body burden might be important for alleviating uremic RLS and further serial TRODAT that correlating RLS with dietary adjustment might be needed.

In this study, we calculated the ratio of dopaminergic neuron uptake depending on the ROI, which was based on the protocol reported by Huang et al. [16]. The protocol has been applied in the diagnosis of Parkinson’s disease and other diseases, such as RLS, which involve dopaminergic transporters. In older people, the dopaminergic uptake may be inferior to that of younger individuals. In our study, the age of patients in the control group was lower than that of patients in the ESRD with and without RLS groups. In contrast to the previous study by Lin et al., the age of the control group was higher than our study (58.25 ± 9.77 years old, vs. 38.85 ± 17.58 years). Therefore, we analyzed the correlation of the total ratio of the dopaminergic neuron uptake and age. The distribution of TRODAT ratio in ESRD with RLS group were below the linear trendline of the control group at caudate nucleus, putamen and striatum. Nevertheless, considering the limited size of the study population, a larger database with a normal population with varying ages might provide us the Tc-99m TRODAT-1 ratio as the reference. Despite several advantages of using TRODAT-1 for diagnosing uremic RLS, our study has several limitations. First, the number of cases was small, with an insufficient number of patients with RLS without renal function impairment or patients with CKD. Second, urinary sampling was limited in patients with ESRD. Third, we did not assess the TRODAT change after target-specific treatment, such as after blood transfusion, enhancing the dosage of erythropoietin or even adding dopaminergic agents, to compare differences in the DAT activity. Therefore, further advanced studies are warranted to help physicians in treating uremic RLS. The kidney handled 32% of the excretion of injected Tc-99m TRODAT-1 within 28 h. Notably, the major organs excreting Tc-99m TRODAT-1 are the liver and biliary tract [41]. Despite the lack of pharmacokinetic studies regarding the use of Tc-99m TRODAT-1 in patients with ESRD, Tc-99m TRODAT-1 might be a safe method for diagnosing RLS. Further applications with CKD patients with RLS might be warrant.

In conclusion, patients with ESRD have a high vulnerability for RLS. In our study, patients with ESRD with RLS had decreased DAT activity based on the Tc-99m TRODAT-1 SPECT functional imaging study. Several contributors, such as anemia, might be the etiology influencing the decreased uptake of dopaminergic neurons. Moreover, elementary heavy metals, such as copper, predicted the incidence of uremic RLS in our study. However, urinary nickel, which correlated negatively with the DAT activity, could be a potential study target to understand the mechanism of RLS or dopaminergic dysregulation in patients with ESRD.

## Figures and Tables

**Table 1:** Demographics of patients. ACEi/ARB: angiotensin-converting enzyme inhibitor/angiotensin receptor blockade. DPP4 inhibitor: dipeptidyl peptidase-4 inhibitor.

	Control Group (*n* = 13)		ESRD without RLS (*n* = 8)		ESRD with RLS (*n* = 11)		
	Number	Percentage	Number	Percentage	Number	Percentage	*p* Value
Age	38.85	17.58	65.25	12.79	57.90	8.99	0.001 *
Male	7	53.85	7	87.5	6	60	0.275
Diabetes mellitus	0	0	4	50	5	50	0.01 *
Medication							
ACEi/ARB	0	0	1	12.5	1	10	0.329
Calcium channel blocker	0	0	2	25	1	10	0.088
Beta-blocker	0	0	2	25	1	10	0.088
Statin	0	0	0	0	1	10	0.276
DPP4 inhibitor	0	0	0	0	1	10	0.276

* *p* < 0.05.

**Table 2:** Hematologic and biochemical results of the three groups. MCV: mean corpuscular volume; GOT: glutamic oxaloacetic transaminase; GPT: glutamic pyruvic transaminase; HbA1c: Glycosylated hemoglobin, type A1C.

	Control Group		ESRD without RLS		ESRD with RLS		
	Mean	SD	Mean	SD	Mean	SD	*p* Value
White blood cell count (/mL	7855.38	1773.38	5783.75	1265.03	7849.00	2435.08	0.045 *
Hemoglobin (g/dL)	14.63	0.73	11.11	0.93	10.91	0.82	0 *
Platelet count (×103/dL)	264.38	54.21	164.50	57.15	197.91	48.37	0.001 *
MCV	84.58	3.08	92.20	4.66	90.03	4.64	0.001 *
Blood urea nitrogen (U/dL)	14.92	4.86	75.75	24.41	73.64	27.43	0 *
Creatinine (mg/dL	0.87	0.21	9.99	2.22	9.45	4.12	0 *
Glomerular filtration rate (mL/min)	97.71	29.20	5.69	1.51	8.94	10.80	0 *
Calcium (mg/dL)	9.18	0.47	9.26	0.76	9.07	1.17	0.881
Phosphorous(mg/dL)	3.94	0.86	5.92	1.34	6.39	2.21	0.002 *
Sodium (mEg/L)	138.67	2.08	138.00	2.45	137.33	2.35	0.672
Potassium (mEq/L)	4.07	0.24	4.74	0.53	4.80	0.85	0.129
Triglyceride	128.75	60.63	99.29	51.57	127.57	66.97	0.584
GOT	61.17	36.20	40.63	57.74	21.56	8.55	0.18
GPT	33.50	30.41	16.00	3.74	21.11	13.82	0.237
uric acid (mg/dL)	5.80	1.36	6.68	1.93	7.54	1.87	0.186
Ferritin (ng/mL)	156.11	117.52	273.31	236.69	314.82	147.12	0.093
intact parathyroid hormone (pg/L)	52.97	22.51	463.85	542.66	311.73	215.06	0.027 *
Transferrin binding capacity (%)	28.71	11.00	26.13	7.35	27.41	7.24	0.825
HbA1c (%)	5.70	0.53	6.56	1.79	7.25	1.59	0.358

* *p* < 0.05.

**Table 3:** TRODAT ratios of the three groups.

	Control Group		ESRD without RLS		ESRD with RLS		
	Mean	SD	Mean	SD	Mean	SD	*p* Value
Caudate, Right	1.47	0.30	1.24	0.24	1.08	0.36	0.022 *
Caudate, Left	1.46	0.30	1.32	0.22	1.11	0.43	0.078
Putamen Right	1.33	0.25	1.18	0.22	1.01	0.39	0.064
Putamen Left	1.35	0.31	1.20	0.18	1.01	0.41	0.071
Basal ganglion, Right	1.40	0.27	1.21	0.22	1.04	0.36	0.011 *
Basal ganglion, left	1.41	0.30	1.26	0.20	1.06	0.41	0.065
Total ratio	1.40	0.28	1.24	0.20	1.05	0.38	0.046 *

* *p* < 0.05.

**Table 4:** Urinary concentration of heavy metals of participants grouped as control, ESRD witht RLS. Cu: Copper. Zn: Zinc. Mn: Manganese. Ni: Nickel. Pb: Lead. Cd: Cadmium. As: Arsenic. Hg: Mercury.

	Control Group		ESRD with RLS		Normal Value	
	Mean	SD	Mean	SD		*p* Value
Cu (μg/dL)	1.67	1.02	4.41	1.92	≤8	0.001 *
Zn (μg/dL)	45.53	29.92	16.45	21.92	15–120	0.033 *
Cu-to-Zn ratio	0.04	0.21	0.60	0.57		0.004 *
Mn (μg/L)	<1.00	0.00	0.93	0.24	≤7.9	0.448
Ni (μg/L)	2.65	1.71	3.21	1.97	≤5.2	0.51
Pb (μg/L)	0.88	0.47	0.60	0.04	≤23	0.086
Cd (μg/L)	0.74	0.52	0.33	0.05	≤2.6	0.11
As (μg/g creatinine)	31.63	18.43	46.23	49.52	<100	0.061
Hg (μg/L)	0.40	0.20	0.97	1.84	≤10	0.775

* *p* < 0.05.

**Table 5:** Correlation of the TRODAT ratio and the biochemical results in the control and ESRD with RLS groups.

	Coefficient of Correlation	*p* Value
BUN	−0.488	0.021 *
Creatinine	−0.492	0.02 *
eGFR	0.525	0.007 *
Hemoglobin	0.531	0.011 *
Intact parathyroid hormone	−0.577	0.015 *
Ferritin	−0.464	0.039 *

* *p* < 0.05.
